# Vector-Enabled Metagenomic (VEM) Surveys Using Whiteflies (Aleyrodidae) Reveal Novel Begomovirus Species in the New and Old Worlds

**DOI:** 10.3390/v7102895

**Published:** 2015-10-26

**Authors:** Karyna Rosario, Yee Mey Seah, Christian Marr, Arvind Varsani, Simona Kraberger, Daisy Stainton, Enrique Moriones, Jane E. Polston, Siobain Duffy, Mya Breitbart

**Affiliations:** 1 College of Marine Science, University of South Florida, Saint Petersburg, FL 33701, USA; ckmarr@mail.usf.edu (C.M.); mya@usf.edu (M.B.); 2 Microbiology and Molecular Genetics, Rutgers, The State University of New Jersey, New Brunswick, NJ 08901, USA; ymseah@eden.rutgers.edu; 3 School of Biological Sciences and Biomolecular Interaction Centre, University of Canterbury, Ilam, Christchurch 8041, New Zealand; arvind.varsani@canterbury.ac.nz (A.V.); simona.kraberger@pg.canterbury.ac.nz (S.K.); daisy.stainton@pg.canterbury.ac.nz (D.S.); 4 Department of Plant Pathology, University of Florida, Gainesville, FL 32611, USA; jep@ufl.edu; 5 Structural Biology Research Unit, Department of Clinical Laboratory Sciences, University of Cape Town, Rondebosch, Cape Town 7701, South Africa; 6 Instituto de Hortofruticultura Subtropical y Mediterránea ‘‘La Mayora’’ (IHSM-UMA-CSIC), Consejo Superior de Investigaciones Científicas, Estación Experimental ‘‘La Mayora’’, Algarrobo-Costa, Málaga 29750, Spain; moriones@eelm.csic.es; 7 Department of Ecology, Evolution and Natural Resources, Rutgers, The State University of New Jersey, New Brunswick, NJ 08901, USA; duffy@aesop.rutgers.edu

**Keywords:** begomovirus, metagenomics, whitefly, vector, ssDNA

## Abstract

Whitefly-transmitted viruses belonging to the genus *Begomovirus* (family *Geminiviridae*) represent a substantial threat to agricultural food production. The rapid evolutionary potential of these single-stranded DNA viruses combined with the polyphagous feeding behavior of their whitefly vector (*Bemisia tabaci*) can lead to the emergence of damaging viral strains. Therefore, it is crucial to characterize begomoviruses circulating in different regions and crops globally. This study utilized vector-enabled metagenomics (VEM) coupled with high-throughput sequencing to survey begomoviruses directly from whiteflies collected in various locations (California (USA), Guatemala, Israel, Puerto Rico, and Spain). Begomoviruses were detected in all locations, with the highest diversity identified in Guatemala where up to seven different species were identified in a single field. Both bipartite and monopartite viruses were detected, including seven new begomovirus species from Guatemala, Puerto Rico, and Spain. This begomovirus survey extends the known diversity of these highly damaging plant viruses. However, the new genomes described here and in the recent literature appear to reflect the outcome of interactions between closely-related species, often resulting from recombination, instead of unique, highly divergent species.

## 1. Introduction

Single-stranded DNA (ssDNA) viruses belonging to the genus *Begomovirus*, the largest of seven genera within the family *Geminiviridae* [1], cause devastating diseases in dicotyledonous plants worldwide. With economic losses estimated on the order of millions of U.S. dollars per year, begomoviruses are a limiting factor in the production of various vegetable, fruit, and fiber crops in subtropical and tropical regions [2,3,4,5]. Moreover, periodic begomovirus epidemics in staple crops, such as cassava, have contributed to widespread famines in the developing world [6].

Begomovirus genomes can be bipartite or monopartite, encoding 5–8 proteins either in two similarly-sized (~2.5–2.7 kb) circular ssDNA molecules known as DNA-A and DNA-B or in a single genomic component (~2.7–2.9 kb) homologous to the DNA-A component of bipartite viruses [7,8]. Monopartite begomoviruses, which are thought to be mainly restricted to the Old World (OW, eastern hemisphere including Africa, Asia, Australia, and Europe), are often accompanied by satellite DNA molecules [9,10]. On the other hand, bipartite begomoviruses are not typically associated with satellite DNAs and can be found in both the New World (NW, western hemisphere including the Americas and Caribbean) and OW, although bipartite viruses are less prevalent than monopartite begomoviruses in the OW [11].

Bipartite begomoviruses encode proteins required for DNA replication, gene regulation, and encapsidation in the genomic DNA-A component, whereas proteins involved in intra- and inter-cellular movement are encoded in the second component, DNA-B [7,8]. The DNA-A component of bipartite begomoviruses and the genomic component of monopartite begomoviruses have ambisense organizations encoding a capsid protein (CP) in the virion-sense strand and the replication-associated protein (Rep), transcriptional activator protein (TrAP), replication enhancer protein (REn), and a virulence factor (C4 or AC4) in the complementary-sense strand. The DNA-A and monopartite genome of OW begomoviruses also encode a precoat protein (PCP) in the virion-sense strand that has not been observed in begomoviruses endemic to the NW. The DNA-B component of bipartite begomoviruses also exhibits an ambisense organization encoding a nuclear shuttle protein (NSP) in the virion-sense strand and a movement protein (MP) in the complementary-sense strand.

The success of begomoviruses as emerging pathogens has been attributed in part to their genomic plasticity, which allows them to adapt to new environments and hosts, as well as the increased prevalence of their highly polyphagous whitefly vector, *Bemisia tabaci* [7,12,13,14]. The high genetic diversity observed in begomoviruses may be explained by their high nucleotide substitution rates which approximate those of RNA viruses [15,16], mechanistic predispositions to recombination processes [17,18,19], and ability to acquire extra DNA components such as satellites [12,20,21]. Recombination processes in particular appear to be a major contributor to begomovirus genetic diversity and adaptability [22,23,24,25,26,27,28,29] and include reassortment of DNA-A, DNA-B, and satellite molecules (*i.e.*, pseudo-recombination), as well as recombination during replication.

The rapid adaptive potential of begomoviruses in novel hosts is exacerbated by the ability of their whitefly vector to exploit agricultural systems. *B. tabaci*, a cryptic species complex comprised of at least 24 morphologically indistinguishable species, is considered among the most damaging agricultural insect pests worldwide and is difficult to control due to its ability to develop insecticide resistance [30,31,32]. *B. tabaci* has a diverse host range that includes more than 600 plant species [33], spanning food and fiber crops as well as cultivated and noncultivated annual and perennial species, and appears to easily spread to new hosts and geographic regions. The polyphagous feeding behavior of *B. tabaci* gives ample opportunities to acquire and transmit a diversity of begomoviruses to new hosts [13,14]. *B. tabaci* may also facilitate the movement of begomoviruses from native, uncultivated vegetation, which may contain new variants and high begomovirus diversity [23,26,34,35], to managed crops leading to new damaging variants [36], as has been observed for other geminiviruses [37].

More exhaustive surveys are needed to increase our knowledge of plant virus diversity, biogeography, evolution, and host range [38]. The genus *Begomovirus* is the most speciose of all currently described viral groups [39,40] and contains many damaging emerging pathogens [12,41], making begomoviruses a prime target group for such surveys. Vector-enabled metagenomics (VEM) [42,43,44], where virions are purified and sequenced directly from insect vectors, is a powerful approach for surveying begomoviruses circulating in a given area. By capturing viruses directly from their whitefly vector, VEM may provide unique insight into begomovirus biogeography and diversity, and serve as a molecular surveillance system capable of recognizing introduced and emerging begomoviruses of agricultural importance. This study coupled VEM with high-throughput sequencing to survey begomoviruses present in whiteflies collected from multiple crops and native vegetation in several countries, allowing the detection of novel species.

## 2. Materials and Methods

### 2.1. Whitefly Collection and Processing for Metagenomic Sequencing

Adult whiteflies were collected from various crop fields and uncultivated native vegetation in four countries (Guatemala, Israel, Spain, and United States) and an island in the Caribbean (Puerto Rico) using battery-operated vacuums and manual aspirators (Table 1). Whiteflies were frozen at −20 °C then equilibrated at room temperature before shipping to the University of South Florida (USA) for processing. Upon receipt, the whiteflies were visually inspected under a dissecting microscope to remove debris and other insects, then stored at −80 °C until further processing.

Virus particles were partially purified from whiteflies before DNA extraction and sequencing. For this purpose, 100–350 whiteflies per field site were homogenized in SM Buffer (50 mM Tris·HCl, 10 mM MgSO_4_, 0.1 M NaCl, pH 7.5) using a bead-beater (BioSpec, Bartlesville, OK, USA) with 1.0 mm glass beads (Research Products International, Mount Prospect, IL, USA) for 1 min. Cells and cellular debris were then removed by filtering through a 0.22 µm Sterivex filter (Millipore, Billerica, MA, USA) and filters were stored at −80 °C. DNA was extracted from 200 µL of filtrate using the QIAmp MinElute Virus Spin Kit (Qiagen, Valencia, CA, USA) following the manufacturer’s instructions and amplified by rolling circle amplification (RCA) using the illustra *TempliPhi* DNA Amplification Kit (GE Healthcare, Little Chalfont, Bukinghamshire, UK) to enrich for small circular templates such as begomovirus genomes [45]. Six replicate RCA reactions were performed for each sample, then the replicates for each sample were pooled and cleaned using the DNA Clean and Concentrator-5 Kit (Zymo Research, Irvine, CA, USA). All RCA products were normalized to 500 ng before library construction and a total of 15 metagenomic libraries were sequenced through multiplexing at a commercial facility using a 454 GS FLX System (Roche, Indianapolis, IN, USA).

### 2.2. PCR Assay to Confirm Whitefly Species and Phylogenetic Group

A PCR assay targeting the mitochondrial cytochrome c oxidase I (mtCOI) gene was performed to confirm the whitefly species processed for VEM. For this purpose, DNA was extracted directly from the 0.22 µm Sterivex filters used to filter whitefly homogenates using the PowerSoil DNA Isolation Kit (MO BIO Laboratories, Inc., Carlsbad, CA, USA) following the manufacturer’s instructions. The mtCOI gene was then amplified using primers designed to distinguish between different whitefly species (BtabUni-F 5′-GAG GCT GRA AAA TTA RAA GTA TTT GG-3′ and BtabUni-R 5′-CTT AAA TTT ACT GCA CTT TCT GCC AYA TTA G-3′) as well as *B. tabaci* phylogenetic groups representing Middle East-Asia Minor 1 (BioB-F 5′-CTA GGG TTT ATT GTT TGA GGT CAT CAT ATA TTC-3′ and BioB-R 5′-AAT ATC GAC GAG GCA TTC CCC CT-3′), Mediterranean (BioQ-F 5′-CTT GGT AAC TCT TCT GTA GAT GTG TGT T-3′ and BioQ-R 5′-CCT TCC CGC AGA AGA AAT TTT GTT C-3′) and New World (BioNW-F 5′-TAC TGT TGR AAT AGA TGT TGA CAC TCG GG-3′ and BioNW-R 5′-GGA AAA AAT GTC AGR TTT ACT CCC WCA AAT ATT-3′) clades [31,46]. Fifty µL PCR reactions contained the following: 1.5 mM MgCl_2_, 1X Apex NH_4_ Buffer, 0.5 µM of each primer, 3% DMSO, 1 µg/µL BSA, 1 U Apex Red Taq DNA Polymerase, and 3 µL of template DNA. Thermocycling conditions consisted of an initial denaturation at 94 °C for 2 min, followed by 35 cycles of 94 °C for 30 s, 46 °C (BtabUni primers) or 64 °C (Bio primers) for 1 min and 72 °C for 1 min, with a final extension at 72 °C for 10 min. Mitochondrial COI gene PCR products obtained with the BtabUni primers were cloned using the CloneJET PCR Cloning Kit (Thermo Scientific, Waltham, MA, USA) and commercially sequenced using vector primers. The different *B. tabaci* phylogenetic groups were distinguished based on positive PCR results for a given “Bio” primer [46].

### 2.3. Metagenomic Data Analysis and Genome Completion

Over 1 million metagenomic reads (average read length 263 nt) were obtained. Sequence reads from each sample were dereplicated using default settings in the CD-Hit web server [47]. Metagenomic reads longer than 100 nt were then assembled with a minimum identity of 98% over 35 nt using Geneious version R7 (Biomatters, Newark, NJ, USA). Both contigs and unassembled reads were compared against the GenBank non-redundant database using BLASTn and BLASTx (*e*-value < 0.001) [48]. BLAST results were summarized and inspected using the Metagenome Analyzer (MEGAN4) software [49] to identify sequences similar to those of begomoviruses. Analyzed contigs and unassembled reads are publicly available on the METAVIR web server (http://metavir-meb.univ-bpclermont.fr/) under project name “Whiteflies” and “Whiteflies_Unassembled”.

Contigs and/or unassembled sequences potentially representing new species based on <91% sequence identity to known begomoviruses [39] were used to design back-to-back (abutting) primers for inverse PCR assays to obtain complete genomes. Inverse PCRs were performed using the HerculaseII Fusion DNA Polymerase (Agilent Technologies, Santa Clara, CA, USA) and products were cloned using the CloneJET PCR Cloning Kit (Thermo Scientific). All genome clones were commercially Sanger sequenced with a minimum of 2× coverage using vector primers and primer walking. Genomes were assembled using the Sequencher software (Gene Codes Corporation, Ann Arbor, MI, USA) and final genome sequences were inspected using SeqBuilder from the Lasergene software package (DNASTAR, Madison, WI, USA). For annotation purposes, ORFs >80 amino acids were compared against the GenBank non-redundant database. Genomes described in this study have been deposited to GenBank with accession numbers shown in Table 2.

### 2.4. Genome Sequence Analysis

#### 2.4.1. Pairwise Comparisons

All pairwise comparisons were performed using the MUSCLE algorithm [50] implemented in the Species Demarcation Tool (SDT) version 1.2 [51].

#### 2.4.2. Phylogenetic Analysis

A maximum likelihood (ML) phylogenetic tree was constructed to evaluate the relationship between a novel virus detected in Spain similar to viruses causing tomato leaf curl disease and sequences from GenBank (*n* = 462). For this purpose, tomato leaf curl viruses (ToLCVs), tomato yellow leaf curl virus (TYLCV), and cassava mosaic viruses full-length DNA-A sequences were aligned using the MUSCLE algorithm [50] implemented in the MEGA5 software [52] and edited manually. A ML phylogenetic tree was then constructed with RAxML v.7.6.3 with the GTRGAMMA substitution model [53], and 1000 bootstrap replicates to assess branch support.

## 3. Results

### 3.1. Overview

This study investigated the diversity of begomoviruses circulating in various crop fields and native vegetation from Guatemala, Israel, Puerto Rico, Spain, and the continental United States (California) (Table 1) through VEM using whiteflies. PCR-based analysis of the mtCOI gene revealed that samples from California, Israel and Puerto Rico contained *B. tabaci* specimens belonging to the Middle East-Asia Minor 1 (MEAM1) clade (formerly known as biotype B), whereas samples from Spain contained specimens from the Mediterranean clade (formerly known as biotype Q) (Table 1). Whiteflies from Guatemala included *B. tabaci* specimens from the MEAM1 and New World clades as well as the greenhouse whitefly *Trialeurodes vaporariorum.*

**Table 1:** Information about whitefly samples used during this study.

Library	Crop/Plant	Location	Collection Date	Whiteflies ^a^
CA_S	Squash	Imperial Valley, California, USA	June 2012	*B. tabaci* (MEAMI)
Guat_T1	Tomato	Salamá, Baja Verapaz, Guatemala	January 2012	*B. tabaci* (NW), *T. vaporariorum*
Guat_T2	Tomato	El Progreso, Jutiapa, Guatemala	January 2012	*B. tabaci* (NW), *T. vaporariorum*
Guat_S	Squash	Teculután, Zacapa, Guatemala	January 2012	*B. tabaci* (MEAM1)
Spain_B	Bean	Torrox, Málaga, Spain	September 2011	*B. tabaci* (MED)
Spain_S	Squash	Torrox, Málaga, Spain	September 2011	*B. tabaci* (MED)
Spain_T	Tomato	Torrox, Málaga, Spain	September 2011	*B. tabaci* (MED)
Spain_W1	European black nightshade	Algarrobo-Costa, Málaga, Spain	September 2011	*B. tabaci* (MED)
Spain_W2	European black nightshade	Algarrobo-Costa, Málaga, Spain	September 2011	*B. tabaci* (MED)
Israel_T	Tomato	Bet Dagan, Israel	March 2011	*B. tabaci* (MEAM1)
Israel_S	Squash	Bet Dagan, Israel	March 2011	*B. tabaci* (MEAM1)
PR_T1	Tomato	Santa Isabel, Puerto Rico	November 2010	*B. tabaci* (MEAM1)
PR_T2	Tomato	Santa Isabel, Puerto Rico	November 2010	*B. tabaci* (MEAM1)
PR_P	Pumpkin	Santa Isabel, Puerto Rico	November 2010	*B. tabaci* (MEAM1)
PR_E	Eggplant	Santa Isabel, Puerto Rico	November 2010	*B. tabaci* (MEAM1)

**^a^** Whitefly species (*Bemisia tabaci* or *Trialeurodes vaporariorum*) used for metagenomic analysis were confirmed through a PCR assay targeting the mitochondrial cytochrome c oxidase I (mtCOI) gene. *B. tabaci* specimens were classified based on mtCOI phylogenetic groups including the Middle East-Asia Minor 1 (MEAM1), Mediterranean (MED), and New World (NW) clades.

Fifteen metagenomic datasets were obtained through pyrosequencing of partially-purified viral nucleic acids from whiteflies. BLAST analysis of assembled contigs and unassembled sequence reads was performed to identify begomoviruses present in each sample. Sequences appearing to represent new species were used to design inverse PCR assays using back-to-back primers to complete and/or confirm begomovirus genomes. New species were identified based on the recently revised begomovirus species demarcation criteria of 91% genome-wide pairwise identity [39,40]. Fifty-three circular genomic components, 40 of which were verified through inverse PCR, were completed (Table 2). These genomic components represent begomovirus complete (DNA-A and DNA-B) or potentially partial (DNA-A) bipartite genomes as well as monopartite genomes. Several begomovirus-associated satellite molecules were also identified; however, these will be described elsewhere.

**Figure 1:**
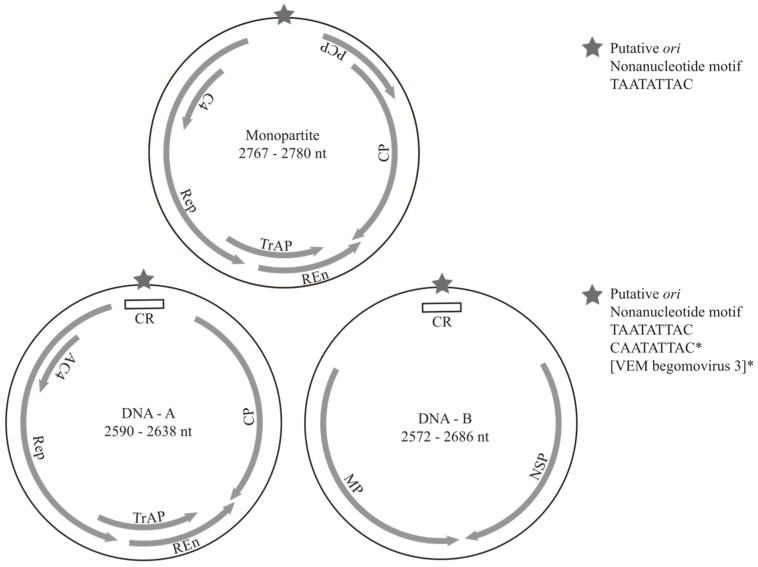
Schematics depicting general genome organization and features observed in novel begomoviruses. All genomes are characterized by a putative origin of replication (*ori*) marked by a stem-loop structure containing a conserved nonanucleotide motif. Begomovirus genomes can be monopartite or bipartite and encode capsid (CP), replication-associated (Rep), transcriptional activator (TrAP), replication enhancer (REn), and virulence factor (C4 or AC4) proteins. Old World begomoviruses also encode a precoat protein (PCP). The DNA-B component of bipartite begomoviruses encodes nuclear shuttle (NSP) and movement protein (MP) proteins.

Complete begomovirus genomes (including both monopartite and bipartite genomes) were identified in each of the samples from various crops and countries; however, novel begomovirus species were only detected in Guatemala, Puerto Rico and Spain (Table 2). Bipartite begomovirus genomes (partial or complete) were recovered from whiteflies collected in all locations with the exception of Spain where only monopartite begomoviruses were identified. Novel species were named as “VEM begomoviruses” since it is not possible to assign a natural host for these viruses. All recovered genomes exhibit typical begomovirus coding capacity and genome organization (Figure 1).

**Table 2:** Begomovirus genomes identified in whiteflies.

Library	Genome ^a^ (Accession)	Best match ^b^ (% Identity)/Accession Number
CA_S	VEM Squash leaf curl virus DNA-A * (KT099117)	SLCV, Imperial Valley, CA (99%)/DQ285016
	VEM Squash leaf curl virus DNA-B * (KT099159)	SLCV, Imperial Valley, CA (95%)/DQ285017
Guat_S	VEM Melon chlorotic leaf curl virus DNA-A * (KT099118)	MCLCuV, Guatemala (96%)/AF325497
	VEM Melon chlorotic leaf curl virus DNA-B * (KT099160)	MCLCuV, Guatemala (96%)/AF325498
	VEM Tomato severe leaf curl virus DNA-A * (KT099119)	ToSLCV, Guatemala and Mexico (97%)/AF130415/DQ347947
	VEM Sida golden mosaic Honduras virus 1a DNA-A (KT099120)	SiGMHV, Honduras (94%)/Y11097
	VEM Sida golden mosaic Honduras virus 1b DNA-A (KT099121)	SiGMHV, Honduras (94%)/Y11098
	VEM Sida golden mosaic Honduras virus 1 DNA-B (KT099161)	SiGMHV, Honduras (82%)/AJ250731
	VEM begomovirus 1a DNA-A (KT099122)	BICV, Mexico (80%)/JX827487
	VEM begomovirus 1b DNA-A (KT099123)	BICV, Mexico (80%)/JX827488
	VEM begomovirus 1c DNA-A (KT099124)	BICV, Mexico (80%)/JX827489
	VEM begomovirus 2 DNA-A (KT099125)	SiYMV, Cuba (84%)/JN411687
	VEM begomovirus 3a DNA-A (KT099126)	CoYSV, Mexico (83%)/DQ875868
	VEM begomovirus 3b DNA-A (KT099127)	CoYSV, Mexico (83%)/DQ875869
	VEM begomovirus 3a DNA-B (KT099162)	SiGMV/SiYVV, Honduras (82%)/AJ250731/Y11100
	VEM begomovirus 3b DNA-B (KT099163)	SiGMV/SiYVV, Honduras (82%)/AJ250731/Y11101
	VEM begomovirus 4 DNA-A (KT099128)	JMV, Dominican Republic and Florida (85%)/KJ174330/KF998097
Guat_T2	VEM Tomato severe leaf curl virus DNA-A * (KT099129)	ToSLCV, Mexico (99%)/DQ347947
	VEM Tomato mosaic Havana virus DNA-A * (KT099130)	ToMHV, Nicaragua (99%)/EF088197
	VEM Tomato mosaic Havana virus DNA-B * (KT099164)	ToMHV, Cuba (92%)/Y14875
Israel_S	VEM Squash leaf curl virus DNA-A (KT099131)	SLCV, Israel (99%)/KM595109
	VEM Squash leaf curl v irus DNA-B * (KT099165)	SLCV, Palestine (99%)/KC441466
	VEM Cotton leaf curl Gezira virus DNA-A (KT099132)	CLCuGV, Egypt and Pakistan (94%)/AF155064/FR751146
PR_T1	VEM Sweet potato leaf curl virus (KT099133)	SPLCV/SPLCLaV, Brazil and Spain (91%)/FJ969833/EU839579
PR_T2	VEM begomovirus 5a DNA-A (KT099134)	WfVEM1/SiGMLV, Florida and Jamaica (90%)/HM859902/HQ009522
	VEM begomovirus 5b DNA-A (KT099135)	WfVEM1/SiGMLV, Florida and Jamaica (90%)/HM859902/HQ009523
	VEM begomovirus 5c DNA-A (KT099136)	WfVEM1/SiGMLV, Florida and Jamaica (90%)/HM859902/HQ009524
	VEM begomovirus 5d DNA-A (KT099137)	WfVEM1/SiGMLV, Florida and Jamaica (90%)/HM859902/HQ009525
	VEM begomovirus 5e DNA-A (KT099138)	WfVEM1/SiGMLV, Florida and Jamaica (90%)/HM859902/HQ009526
	VEM begomovirus 5 DNA-B (KT099166)	SiYVV, Cuba (79%)/HE806449
	VEM begomovirus 6b DNA-B (KT099167)	SiGMFV, Florida (78%)/HE806443
PR_P	VEM begomovirus 6 DNA-A (KT099139)	SiYMV, Cuba (88%)/HQ822123
	VEM begomovirus 6a DNA-B (KT099168)	SiGMFV, Florida (78%)/HE806443
	VEM Sweet potato leaf curl virus (KT099140)	SPLCV, Puerto Rico (93%)/DQ644563
	VEM Macroptilium mosaic Puerto Rico virus DNA-A* (KT099141)	MaMPRV, Puerto Rico (99%)/AF449192
	VEM Macroptilium mosaic Puerto Rico virus DNA-B* (KT099169)	MaMPRV, Puerto Rico (98%)/AY044134
PR_E	VEM begomovirus 5f DNA-A (KT099142)	WfVEM1/SiGMLV, Florida and Jamaica (90%)/HM859902/HQ009522
	VEM Sweet potato leaf curl virus (KT099143)	SPLCV/SPLCLaV, Brazil, Puerto Rico and Spain (91%)/FJ969833/DQ644563/EU839579
Spain_B	VEM Sweet potato leaf curl virus 1 (KT099144)	SPLCV, Spain (97%)/EU856364
Spain_S	VEM Sweet potato leaf curl virus 2 (KT099145)	SPLCESV, Spain (94%)/EF456743
Spain_T	VEM begomovirus 7a (KT099146)	ToLCNamV, Madagascar (81%)/AM701764
	VEM begomovirus 7b (KT099147)	ToLCNamV, Madagascar (81%)/AM701765
	VEM begomovirus 7c (KT099148)	ToLCNamV, Madagascar (81%)/AM701766
	VEM begomovirus 7d (KT099149)	ToLCNamV, Madagascar (81%)/AM701767
	VEM begomovirus 7e (KT099150)	ToLCNamV, Madagascar (81%)/AM701768
	VEM begomovirus 7f (KT099151)	ToLCKMV, Comoros (82%)/AM701759
	VEM begomovirus 7g (KT099152)	ToLCKMV, Comoros (81%)/AM701759
Spain_W1	VEM begomovirus 7h (KT099153)	ToLCNamV, Madagascar (81%)/AM701764
	VEM begomovirus 7i (KT099154)	ToLCNamV, Madagascar (81%)/AM701765
	VEM begomovirus 7j (KT099155)	ToLCNamV, Madagascar (81%)/AM701766
	VEM begomovirus 7k (KT099156)	ToLCNamV, Madagascar (81%)/AM701767
	VEM Tomato yellow leaf curl virus-[Almeria] 1 * (KT099157)	TYLCV, Spain (99%)/AJ489258
Spain_W2	VEM Tomato yellow leaf curl virus-[Almeria] 1a* (KT099158)	TYLCV, Spain (99%)/AJ489258

^a^ Genomes detected in whiteflies were identified as “VEM” followed by the virus name with the best match in the database. However, genomes from novel species that shared <91% pairwise identity with sequences found in GenBank were named as “VEM begomovirus #”. Percent nucleotide identities were calculated across entire genomes only. Genomes that were not PCR-verified are highlighted with an asterisk (*); ^b^ Virus names for best matches in GenBank are abbreviated according to underlined letters in the “Genomes” column. Best matches for novel species identified in this study include Blechum interveinal chlorosis virus (BICV), Sida yellow mottle virus (SiYMV), Corchorus yellow spot virus (CoYSV), Sida golden mosiac virus (SiGMV), Sida yellow vein virus (SiYVV), Jatropha mosaic virus (JMV), Whitefly VEM 1 begomovirus (WfVEM1), Sida golden mosaic Liguanea virus (SiGMLV), Sida golden mosaic Florida virus (SiGMFV), Tomato leaf curl Namakely virus (ToLCNamV), and Tomato leaf curl Comoros virus (ToLCKMV).

### 3.2. Bipartite Begomoviruses

Thirteen distinct begomovirus genomes, most likely representing bipartite viruses, were identified. The majority of these genomes were detected in whitefly samples collected from the NW. Both genomic components, DNA-A and DNA-B, were recovered for eight of the genomes. These bipartite genomes exhibited general genomic features found in bipartite begomoviruses (Supplemental Table S1), including a putative tyrosine phosphorylation site ([R/K]X_2,3_[D/E]X_2,3_Y) recently identified in the movement protein (MP) of NW bipartite begomoviruses [54]. The DNA-As of the remaining five genomes detected in the NW for which only one component was retrieved also exhibited characteristics suggesting that they represent one component of a bipartite begomovirus (as opposed to a monopartite virus). In addition to nucleotide identities to known bipartite begomovirus genomes, the identified DNA-A components exhibit an average genome size of 2614 nts (range 2590–2638 nts) and lack an ORF encoding for a PCP, both of which are typical of NW bipartite begomoviruses. All but one of the DNA-A components contained a distinctive N-terminal motif (PWRsMaGT) in their CP [55], which is typical of DNA-A components from bipartite genomes (Supplemental Table S1). However, one DNA-A identified in the NW, VEM begomovirus 1, had a motif containing several amino acid substitutions that have not been previously observed (PWR_L_VETL).

The lowest begomovirus diversity was detected in whiteflies from California (CA_S), where only squash leaf curl virus (SLCV) was detected (Table 2). The SLCV genome (DNA-A and DNA-B) was 99% identical to a SLCV genome previously reported from the same region. SLCV was also identified in whiteflies collected from squash in Israel. The Israel SLCV genome (DNA-A and DNA-B) was 99% identical to the genome of a strain previously identified in Israel and neighboring countries including Jordan, Lebanon, and Palestine [56]. The SCLV genomes from California and Israel shared 97.8% and 94.8% genome-wide pairwise identity among the DNA-A and DNA-B components, respectively.

The highest diversity of bipartite begomoviruses was found in the whiteflies collected from squash in Guatemala. Nine begomovirus genomes were recovered from Guatemala, seven of which were from whiteflies collected in a single squash field site, including four novel species named VEM begomovirus 1 through 4 (Table 2). VEM begomovirus 1 and 3 are related to viruses previously reported from Central America, whereas VEM begomovirus 2 and 4 are related to viruses reported from the Caribbean. Interestingly, VEM begomovirus 3 had a unique nonanucleotide motif (CAATATTAC) at the putative origin of replication (*ori*) of both genomic components compared to the TAATATTAC motif conserved in the majority of known begomoviruses (Figure 1) [8,57]. Although it has been shown that replication initiation may not be restricted to the canonical nonanucleotide motif TAATATTAC, changes to this nonamer may significantly reduce the efficiency of the replication initiation reaction where Rep introduces a nick between nucleotide positions +7 and +8 of the nonamer [58]. The discovery of begomovirus genomes, such as VEM begomovirus 3 and corchorus golden mosaic virus [11], with nonamers that deviate from the canonical begomovirus formula suggests that there are isolates that may employ slightly different replication strategies or exhibit commensurate functional changes in the Rep to compensate for reaction efficiency.

Two additional novel begomovirus species, VEM begomovirus 5 and 6, were identified in Puerto Rico (Table 2). VEM begomovirus 5 was identified in whiteflies from two field sites and is closely related (90% genome-wide pairwise identity) to viruses identified in Florida and Jamaica. Six VEM begomovirus 5 genomes sharing more than 98% identity to each other were recovered. VEM begomovirus 6 was also identified in two field sites and is closely related to viruses identified in Cuba (88% genome-wide pairwise identity) and Guatemala (85% genome-wide pairwise identity to VEM begomovirus 2 identified here). In addition, a macroptilium mosaic Puerto Rico virus was identified in one of the sites, which shares 99% and 98% genome-wide pairwise identity with the DNA-A and DNA-B genomic sequences, respectively, of an isolate previously reported from the island [59].

### 3.3. Monopartite Begomoviruses

Monopartite begomoviruses were detected in whiteflies from Israel, Puerto Rico, and Spain (Table 2). The Israel squash (Israel_S) dataset contained contig sequences that shared >98% identity with hollyhock leaf crumple virus (HLCrV) and cotton leaf curl Gezira virus (CLCuGV) genomes, whereas the Israel tomato dataset only exhibited the presence of tomato yellow leaf curl virus (TYLCV) (data not shown). All of these monopartite viruses have been previously reported from the area. A CLCuGV genome that shares 94% genome-wide pairwise identity with genomes from a strain reported from Egypt, Jordan and Pakistan was retrieved from the Israel_S dataset.

Viral sequences similar to those of monopartite sweet potato-infecting viruses (sweepoviruses) were detected in Puerto Rico and Spain. Sweet potato leaf curl virus (SPLCV) genomes were recovered from three of the Puerto Rico sampling sites. These viruses, named VEM Sweet potato leaf curl virus [Puerto Rico], shared more genomic similarities to each other than to known SPLCV genomes, including those previously reported from the island. VEM SPLCV genomes shared 92%–96% identity among themselves, and 91%–93% identity with sequences in the database, including SPLCV and Sweet potato leaf curl Lanzarote virus genomes reported from Brazil, Puerto Rico, and Spain (Table 2). Sweepovirus sequences were also detected in the three metagenomic datasets from Spain that were prepared from whiteflies collected from crops; however, sweepovirus sequences were not detected in whiteflies collected from weeds. Two sweepovirus genomes identified in this study, VEM Sweet potato leaf curl virus 1 and VEM Sweet potato leaf curl virus 2, were closely related to sweepovirus species previously reported from Spain (Table 2). The VEM SPLCV 1 genome shares 97% identity with a SPLCV genome, whereas the latter shares 94% identity with a Sweet potato leaf curl Spain virus (SPLCESV) genome.

**Figure 2:**
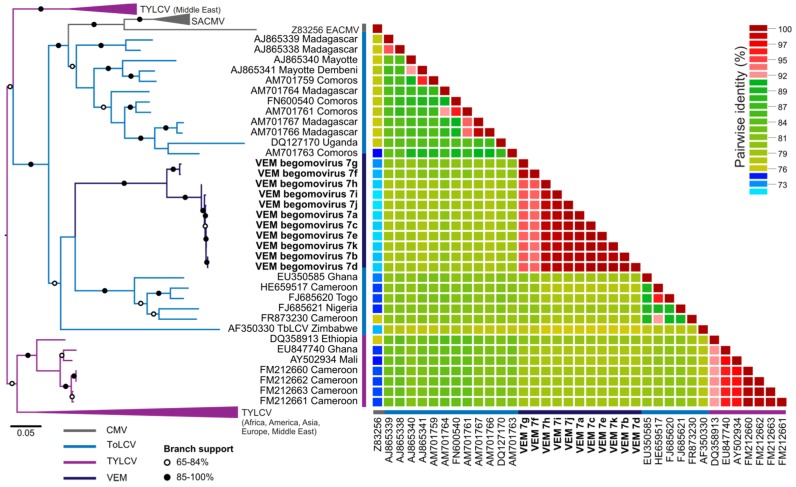
Maximum likelihood (ML) phylogenetic tree (**left**) and two-dimensional color-coded matrix depicting genome-wide pairwise identities (**right**) among genomes related to tomato leaf curl virus (ToLCV) detected through the vector-enabled metagenomic (VEM; dark blue) approach used in this study and related sequences. The ML tree includes sequences representing ToLCV (blue), tomato yellow leaf curl virus (TYLCV; purple), and cassava mosaic viruses (CMVs; grey). CMVs include South African cassava mosaic virus (SACMV) and East African cassava mosaic virus (EACMV). Branches with bootstrap support >85% are indicated with black circles, whereas branches exhibiting 60%-84% support are marked with a white circle. A list of sequences used for phylogenetic analysis is provided in Supplemental File S2. Genome-wide pairwise identities are shown for viral genomes related to ToLCV identified through VEM as well as the most closely-related sequences from Africa and the South-West Indian Ocean (SWIO) islands.

Monopartite begomoviruses that presumably infect tomatoes were also detected in samples from Spain. A TYLCV variant known to be present in Spain was detected in all metagenomic datasets from Spain (data not shown). However, three out of five datasets also exhibited the presence of a divergent genome most similar to sequences from monopartite viruses causing tomato leaf curl disease identified in the South-West Indian Ocean (SWIO) islands, including tomato leaf curl Namakely virus and tomato leaf curl Comoros virus. The eleven genomes recovered for this virus share 93%–99% genome-wide pairwise identity, thus representing a single species named here VEM begomovirus 7. Since SWIO island begomoviruses have been found to be related to tomato and cassava-infecting viruses from Africa and the Mediterranean [27,60,61], genome-wide pairwise identities and phylogenetic relationships among these viruses and VEM begomovirus 7 were evaluated (Figure 2). The VEM begomovirus 7 genomes have overlapping identity ranges with genomes from tomato-infecting viruses isolated from African countries bordering the Gulf of Guinea (Ghana, Togo, Cameroon, and Nigeria; 77%–80% identity), Uganda (78%–79% identity) and SWIO islands (Comoros, Madagascar, Mayotte; 78%–81% identity). In addition, VEM begomovirus 7 genomes shared similar identity ranges with genomes of South African cassava mosaic virus (SACMV) (South Africa and Madagascar; 76%–80% identity). Despite top BLAST matches and overlapping pairwise identities, phylogenetic analysis suggests that VEM begomovirus 7 is more closely related to tomato-infecting viruses identified in African countries bordering the Gulf of Guinea and Zimbabwe (Figure 2).

## 4. Discussion

### 4.1. Diversity Revealed by VEM

This study implemented the VEM approach to survey begomoviruses directly from whiteflies found in crop fields and weeds located in different countries. The VEM survey captured the diversity of begomoviruses that were in transit between hosts in all of the investigated locations, including seven novel virus species. Notably, as observed in the dataset from Guatemala squash, up to seven distinct begomovirus species were found circulating in a given area. To our knowledge, previous large scale surveys of visibly infected crops or weeds that have implemented RCA coupled with restriction enzyme digests or conducted high throughput sequencing of RCA products have reported only between one and five begomovirus species at a single time (e.g., [35,36,62]). Although surveys of visibly infected plants are a critical component of agricultural surveillance, VEM-based approaches can complement these efforts as a less labor-intensive method for recovering a broader swath of begomovirus diversity. Such approaches also provide insight into the reservoir of viral genetic diversity that is often overlooked due to the lack of visible symptoms.

VEM can pinpoint the presence of a viral species at a given time; however, this method does not provide information regarding the transmissibility of a given virus by whiteflies. Most begomovirus species are known to be transmitted by *B. tabaci*; however, there are some isolates which have lost their ability to be vector-transmitted but may still be acquired by *B. tabaci* (e.g., abutilon mosaic virus and honeysuckle yellow vein virus [63,64,65]). Moreover, two of the samples from Guatemala contained a mixture of both *B. tabaci* and *T. vaporariorum* since both species can be found together in fields despite having different population dynamics [66,67]. Although *T. vaporariorum* can accumulate begomoviruses, this whitefly species is not generally known as a begomovirus vector [68]. Nevertheless, the VEM effort provided a glimpse of begomovirus species present in the field regardless of their vector-transmissibility and, more importantly, provided a window into potential viral interactions that may occur in the field.

Since the VEM approach provides a strategy for “unbiased” sampling based on the ability of whiteflies to accumulate begomoviruses, we expected to retrieve numerous novel species. The current species demarcation criteria (91% cut off) for members of the genus *Begomovirus* were chosen based on true pairwise comparisons of 3123 full-length begomovirus DNA-A genomes [39]. Further examination of the data used to establish the new species demarcation criteria shows that approximately 1% or less of the DNA-A pairwise-comparisons performed display >80% pairwise identity, while the highest proportion of pairwise identities range between 64% and 77%. While seven novel begomovirus species were identified from Guatemala (*n* = 4), Puerto Rico (*n* = 2) and Spain (*n* = 1), it is notable that each of these novel VEM species shares >80% genome-wide identity with known begomovirus genomes. Similarly, in a survey of peer-reviewed articles describing novel begomovirus species between January 2011 and March 2015 (*n* = 31), only a single reported genome, representing hemidesmus yellow mosaic virus [69], shares less than 70% identity with genomes from known species. The vast majority (81%) of the reported genomes from novel species share ≥80% pairwise identity or between 77% and 79% pairwise identity (16% of the reports) with sequences in the database. Even amongst wild or native vegetation, which is significantly understudied compared to crops, 67% of the begomovirus genomes reported from non-cultivated hosts (*n* = 9) share >80% identity to genomes from described species. Therefore, it appears that most of the newly captured begomovirus diversity reflects the outcome of interactions between closely-related species, often resulting from recombination events, instead of unique, highly divergent species.

The increased recovery of closely-related recombinant genomes reflects the propensity of begomoviruses to recombine, which may increase their evolutionary potential and adaptability to new hosts and habitats [25,28,70,71]. One proposed mechanism for the emergence of new begomoviruses is the movement of viruses between cultivated hosts and non-cultivated hosts, which may harbor higher genetic diversity and serve as perennial hosts [5,23]. It has been recognized that infection of non-cultivated plant species leads to higher levels of standing genetic variability and that recombination, rather than adaptive selection, is the driving force behind higher begomovirus diversity in non-cultivated hosts [23]. This study supports the view that many newly described begomoviruses represent variations of known genomic themes. Describing and monitoring these new species variants in both uncultivated and cultivated vegetation is critical since they may find a successful niche in agricultural regions, allowing them to emerge as economically significant pathogens.

### 4.2. Begomovirus Biogeography

Consistent with current knowledge regarding begomovirus biogeography, most of the begomoviruses detected through VEM in the NW were bipartite whereas most of the begomoviruses detected in the OW were monopartite. The only bipartite genome detected in samples from the OW, specifically Israel, was SLCV (which was also detected in samples from California). SLCV is known to have been introduced to the Middle East from the NW [72,73] and the low genetic variation among NW and OW SLCV isolates has been attributed to low levels of recombination for this virus and indicates that SLCV easily migrated into the OW [56].

The VEM approach allowed the recovery of both DNA-A and DNA-B components of several bipartite viruses in the NW. The genome size for all the components (Supplemental Table S1) fell within average genome sizes for NW bipartite viruses, which on average have reduced genome sizes compared to their OW counterparts [54]. In addition to this size reduction, the MPs of the NW DNA-B components are under stronger purifying selective pressure than those of the OW viruses [54]. This stronger selection appears to maintain a putative tyrosine phosphorylation site found in the MP encoded by most NW DNA-B components [54], including all of the DNA-B components detected in this study (Supplemental Table S1). However, NW DNA-A and DNA-B components seem to be under different evolutionary pressures, with the DNA-B exhibiting more overall genetic diversity than the DNA-A [74]. For several bipartite genomes detected in this study the DNA-B component was less similar to the DNA-B of previously characterized genomes than the DNA-A component, which exhibited higher identities to the DNA-A of known species (Table 2). Therefore, although the genes encoded by DNA-B of NW viruses seem to be under more selective pressure compared to their OW counterparts [54], this component may still experience different evolutionary histories compared to its cognate DNA-A within the NW.

Global trade has been identified as one of the factors driving begomovirus evolution since it provides the opportunity for species to reach new territories and adapt to new hosts, as well as introduces vectors with different host preferences [12]. The VEM approach used here resulted in the detection of monopartite begomovirus genomes which reflect interactions among begomoviruses from distant countries. Sweepoviruses, a unique group of monopartite begomoviruses that clusters separately from both NW and OW begomoviruses [75], were detected in libraries from Spain and Puerto Rico. All the detected sweepovirus genomes were similar to viral genomes previously reported from their respective areas. However, recombination analyses suggest that the SPLCV genomes detected here are the result of recombination events among isolates from Brazil, China, Puerto Rico, and Spain [76]. The extensive recombination events that have been documented for sweepoviruses [75,76] complicate species assignment following current taxonomic criteria. Based on phylogenetic analyses and the distribution of pairwise identities among sweepovirus genomes it was recently proposed that SPLCESV and IYVV should be merged under the SPLCV species [39], increasing the within-species diversity of SPLCV [76]. Therefore the genome from Spain identified here that is similar to a SPLCESV genome has been named VEM SPLCV.

In addition, VEM begomovirus 7, with a divergent genome most similar to genomes from viruses causing tomato leaf curl disease, was detected in samples from Spain. Notably, VEM begomovirus 7 genomes share higher identity with genomes from tomato-infecting monopartite viruses from SWIO islands, which have been found to be evolving in isolation [27,60], compared to Mediterranean viruses. Studies investigating indigenous SWIO island monopartite tomato and tobacco-infecting begomoviruses have shown that they are more closely related to African cassava begomoviruses than to Mediterranean and African tomato begomoviruses [27,60,61]. Despite similarities to genomes from SWIO island viruses and detection in samples from Spain, phylogenetic analysis suggests that VEM begomovirus 7 is more closely related to African tomato-infecting viruses rather than cassava mosaic viruses or Mediterranean viruses. It is clear that there is high genetic exchange among African, SWIO Island, and Mediterranean viruses. Further sampling may recover missing evolutionary links among these viruses and may provide insights into virus movements over this large geographic area (e.g., [77]).

Recent studies have uncovered an indigenous monopartite virus from the NW, tomato leaf deformation virus (ToLDeV), highlighting that monopartite viruses have not only evolved in the OW [78,79]. Samples from Guatemala revealed the presence of four distinct DNA-A genomes with characteristics similar to those found in the DNA-A of bipartite begomoviruses. However, no cognate DNA-B components were identified for any of these genomes, making it difficult to identify with certainty if these DNA-A components represent bipartite or monopartite viruses. One of these genomes resembles a currently classified species, *Tomato severe leaf curl virus*, for which no DNA-B has been reported. The lack of a cognate DNA-B component is further complicated by the fact that the monopartite ToLDeV exhibits all the genomic characteristics that are typical of NW bipartite begomoviruses [78,79]. These features include genome size, the absence of a PCP, and the presence of a unique motif on the N-terminus of the CP. All of the VEM DNA-A genomes described here exhibit all those characteristics except for VEM begomovirus 1 which exhibits a variation of the canonical NW CP motif (Supplemental Table S1). It has been suggested that bipartite begomoviruses evolved from monopartite viruses either by duplication of the DNA-A component and acquisition of a movement gene or through a satellite that was captured and “domesticated” by DNA-A into DNA-B [74,80,81]. The discovery of ToLDeV indicates a new evolutionary direction since evidence suggests that it evolved from a bipartite virus through recombination and convergent evolution [78] showing gene reduction rather than expansion as has been generally hypothesized. Further experiments are needed to assess if the DNA-A components detected here indeed represent other NW monopartite viruses, which would suggest that begomovirus genome reduction is a more widespread phenomenon in the NW, or if they represent bipartite viruses whose cognate DNA-B components were simply not identified in our survey.

## Supplementary Files

Supplementary File 1
