# Supplementary material for: Vector-Enabled Metagenomic (VEM) Surveys Using Whiteflies (Aleyrodidae) Reveal Novel Begomovirus Species in the New and Old Worlds
Source: Viruses. 2015 Oct 26;7(10):5553–70. doi: 10.3390/v7102895 (PMC4632403; doi:10.3390/v7102895)
Supplement: Supplementary File 1 [file viruses-07-02895-s001.zip › Supplemental File S2.pdf]

Supplemental File S2. Sequences used for phylogenetic analysis and genome-wide pairwise comparisons displayed in Figure 2.

>AB014346_Japan
>AB014347_Japan
>AB110217_Japan_Nagasaki_Omura
>AB110218_Japan_Shizuoka_Shimizu
>AB116629_Japan__Miyazaki_Miyazaki
>AB116631_Japan__Kumamoto_Misumi
>AB116632_Japan__Shizuoka_Yaizu
>AB116633_Japan__Aichi_Atumi
>AB116634_Japan__Mie_Kisozaki
>AB116635_Japan__Shizuoka_Daito
>AB116636_Japan__Shizuoka_Osuka
>AB192965_Japan__Kochi_Tosa_Usa
>AB363566_Japan_Okinawa_Tomigusuku_Zayasu
>AB439841_Japan__Tochigi_Oyama
>AB439842_Japan__Aichi_Tokai
>AB613208_South_Korea_Iksan
>AB613209_South_Korea_Nonsan
>AB636264_South_Korea_Yesan_city
>AB636409_South_Korea_Gangju_city
>AB636410_South_Korea_Cheju_city
>AB636411_South_Korea_Cheju_city
>AB636412_South_Korea_Cheju_city
>AB669434_South_Korea
>AB921568_Japan__Osaka_Takatsuki
>AF024715_Dominican_Republic
>AF071228_Spain
>AF105975_Portugal
>AF271234_Spain
>AF350330_TbLCV_ZimbabweVirus
>AJ132711_Iran
>AJ223505_Cuba
>AJ422132_SACMV_Madagascar_M12
>AJ489258_Spain_Almeria
>AJ519441_Spain_Andalucia
>AJ519675_Spain_Canary_Islands_Gran_Canaria_Tirajana
>AJ812277_Turkey_Mersin
>AJ865337_Reunion
>AJ865338_Madagascar_Morondova_2001
>AJ865339_Madagascar_Toliary
>AJ865340_Mayotte_Kahani

>AJ865341_Mayotte_Dembeni
>AM282874_China_Shanghai
>AM409201_Reunion_SaintGilles_les_Hauts
>AM698117_China_Zhejiang_Province
>AM698118_China_Zhejiang_Province
>AM698119_China_Zhejiang_Province
>AM701759_Comoros_Anjouan_Bambas
>AM701761_Comoros_Grande_Comore_Dimadjou
>AM701763_Comoros_Moheli_Fomboni
>AM701764_Madagascar_Antsiranana_Namakely
>AM701766_Madagascar_Diana_Antsalaka
>AM701767_Madagascar_Menabe_Miandrivazo
>AY044138_Sudan
>AY134494_Puerto_Rico
>AY227892_Spain
>AY502934_Mali
>AY530931_USA__Florida
>AY594174_Egypt__Ismailia_Governate
>DQ127170_Uganda__Iganga
>DQ144621_Italy__Sicily
>DQ358913_Ethiopia__Melkassa
>DQ631892_Mexico__Culiacan
>EF051116_Lebanon
>EF054893_Jordan
>EF054894_Jordan
>EF060196_Morocco__Berkane_northeast_region
>EF110890_USA__Texas
>EF110891_Yemen
>EF158044_Jordan
>EF185318_Lebanon
>EF433426_Jordan
>EF523478_Mexico
>EF539831_USA__Brawley_California
>EU031444_China__Shanghai
>EU085423_Iran__BandarAbbas
>EU143745_Jordan__Homrat_AlSahen
>EU350585_Ghana_Akumadan
>EU635776_Iran__Kerman_Kahnooj
>EU847740_Ghana__Kumasi
>FJ355946_Iran
>FJ439569_Netherlands
>FJ609655_Mexico__Juan_Jose_Rios_Guasave_Sinaloa
>FJ685620_Togo

>FJ685621_Nigeria
>FJ956701_Oman
>FJ956702_Oman
>FJ956703_Oman
>FJ956704_Oman
>FJ956705_Oman
>FM212660_Cameroon_North_Province
>FM212661_Cameroon_North_Province
>FM212662_Cameroon_North_Province
>FM212663_Cameroon_North_Province
>FN252890_China_Zhejiang_province
>FN256256_China_Zhejiang_province
>FN256257_China_Shandong_province
>FN256258_China_Shanghai
>FN256259_China_Jiangsu_province
>FN600540_Comoros_Grande_Comore_Island_Foubouni
>FN650808_China_Anhui
>FR851297_Grenada_Hermitage
>FR873230_Cameroon_South_Western
>GQ141873_South_Korea__Busan
>GQ352537_China
>GQ352538_China
>GQ861426_Jordan
>GQ861427_Jordan
>GU076440_Iran
>GU076441_Iran
>GU076442_Iran
>GU076443_Iran
>GU076444_Iran
>GU076445_Iran
>GU076446_Iran
>GU076447_Iran
>GU076448_Iran
>GU076449_Iran
>GU076450_Iran
>GU076451_Iran
>GU076452_Iran
>GU076453_Iran
>GU076454_Iran
>GU111505_China
>GU199587_China
>GU322423_USA__Hawaii
>GU322424_USA__Hawaii

>GU325632_South_Korea__Jeju_Island
>GU325633_South_Korea__Jeju_Island
>GU325634_South_Korea__Boseong
>GU348995_China__Hebei
>GU355941_Guatemala__Salama_Valley
>GU434141_China__Shanghai_Minhang
>GU434142_China__Anhui_Province_Hefei
>GU434144_China__Shanghai
>GU563330_China__Tianjin
>GU951436_China__Baoding_City
>GU951437_China__Handan_City
>GU983859_China__Beijing
>HE603241_New_Caledonia
>HE603242_New_Caledonia
>HE603243_New_Caledonia
>HE603244_New_Caledonia
>HE603245_New_Caledonia
>HE603246_New_Caledonia
>HE659517_Cameroon_South_WestFontem
>HE819240_Oman_AlKhaburah
>HE819241_Oman_Bidiya
>HE819242_Oman_Nizwa
>HE819243_Oman_Bid_Bid
>HE819245_Oman
>HM043732_China__Shouguang_of_Shandong_province
>HM130913_South_Korea__Jeonju
>HM130914_South_Korea__Jeju
>HM208334_China__Hebei
>HM358879_China__Yinan_Shandong_Provinces
>HM448447_Mauritius
>HM459851_Mexico__Baja_California_Sur
>HM627880_China__Dezhou_Shandong
>HM627881_China__Dongying_Shandong
>HM627882_China__Heze_Shandong
>HM627883_China__Jinan_Shandong
>HM627884_China__Laiyang_Shandong
>HM627885_China__Zibo_Shandong
>HM856873_South_Korea
>HM856909_South_Korea__Busan
>HM856910_South_Korea__Buyeo
>HM856911_South_Korea__Goseong
>HM856912_South_Korea__Gunwi
>HM856913_South_Korea__Gwangju

>HM856914_South_Korea__Gwangyang
>HM856915_South_Korea__Gyeongju
>HM856916_South_Korea__Iksan
>HM856917_South_Korea__Jangheung
>HM856918_South_Korea__Nonsan
>HM856919_South_Korea__Uiseong
>HM988987_USA
>HQ260984_South_Korea__Chang_won
>HQ702861_China__Linzi_Shandong
>HQ702862_China__Shouguang_Shandong
>HQ702863_China__HeZe_Shandong
>JF301667_China__Jining_Shandong_province
>JF301668_China__Qingdao_Shandong_province
>JF414236_China__Taian_of_Shandong_province
>JF414237_China__Henan
>JF451352_Kuwait
>JF727878_China__Shijiazhuang_Hebei_province
>JF817218_China__Shijiazhuang_Hebei_province
>JF833036_China__Langfang_Hebei_province
>JF964959_China__Quzhou_Handan_Hebei_province
>JN183873_South_Korea__Damyang
>JN183874_South_Korea__Damyang
>JN183875_South_Korea__Damyang
>JN183876_South_Korea__Damyang
>JN183877_South_Korea__Gongju
>JN183878_South_Korea__Okcheon
>JN183879_South_Korea__Andong
>JN412854_China__Shanxi_Province
>JN604484_Oman
>JN604485_Oman
>JN604486_Oman
>JN604487_Oman
>JN604488_Oman
>JN680149_South_Korea
>JN680353_Mexico
>JN859134_Portugal
>JN859135_Portugal
>JN859136_Portugal
>JN859137_Portugal
>JN859138_Portugal
>JN990922_China__Baoding_of_Hebei_Province
>JN990923_China__Langfang_of_Hebei_Province
>JN990924_China__Hebi_Junxian_of_Henan_Province

>JN990925_China__Puyang_of_Henan_Province
>JN990926_China__Zhengzhou_of_Henan_Province
>JN990927_China__Jinan_Shandong
>JN990928_China__Laiwu_of_Shandong_Province
>JQ004028_China__Henan_Zhengzhou
>JQ004045_China__Baoding_Dingzhou_Hebei_province
>JQ004046_China__Cangzhou_Qingxian_Hebei_province
>JQ004047_China__Hengshui_Raoyang_Hebei_province
>JQ004048_China__Xinxiang_Huojia_Henan_province
>JQ004049_China__Kaifeng_Henan_province
>JQ004050_China__Puyang_Hebei_province
>JQ004051_China__Zhengzhou_Shangjie_of_Shandong_province
>JQ004052_China__Jiaozuo_Xiuwu_Henan_province
>JQ013089_South_Korea
>JQ013090_South_Korea
>JQ013091_South_Korea
>JQ034613_China__Henan_Province_Anyang_Henan_province
>JQ038232_China__Yongqing_Hebei_province
>JQ038233_China__Macun_Henan_province
>JQ038234_China__Qingfeng_Henan_province
>JQ038235_China__Qixian_Henan_province
>JQ038236_China__Tangyin_Henan_province
>JQ038237_China__Weihui_Henan_province
>JQ038238_China__Wuzhi_Henan_province
>JQ038239_China__Xinxiang_Henan_province
>JQ038240_China__Changle_Shandong_province
>JQ231214_Iran
>JQ303121_Mexico
>JQ326957_China
>JQ354991_Iraq
>JQ414025_Iran
>JQ807735_China__Kashi_Xinjiang
>JQ867092_China
>JQ928346_Iran__Birjand_South_Khorasan_province
>JQ928347_Iran
>JQ928348_Iran__Dargaz_Khorasan_Razavi_province
>JX070042_China__Jinan_Shandong
>JX070043_China
>JX070044_China__Linyi_Shandong
>JX070045_China__Heze_Shandong
>JX131286_Jordan
>JX444575_Jordan
>JX456637_China

>JX456638_China
>JX456639_China
>JX456640_China
>JX456641_China
>JX456642_China
>JX456643_China
>JX456644_China
>JX669541_China__Cangshan_Shandong_province
>JX669542_China__Feixian_Shandong_province
>JX669543_China__Mengyin_Shandong_province
>JX669544_China__Yantai_Shandong_province
>JX675237_China
>JX856172_China__Taian_Shandong_province
>JX856173_China__Tengzhou_Shandong_province
>JX910534_China
>JX961665_South_Korea
>JX961666_South_Korea
>JX961667_South_Korea
>JX961668_South_Korea
>JX961669_South_Korea
>KC106635_Iran__Bojnurd
>KC106636_Iran__Bojnurd
>KC106637_Iran__Bojnurd
>KC106638_Iran__Bojnurd
>KC106641_Iran__Bojnurd
>KC106643_Iran__Bojnurd
>KC106645_Iran__Bojnurd
>KC106646_Iran__Bojnurd
>KC106648_Iran__Bojnurd
>KC106649_Iran__Bojnurd
>KC106650_Iran__Bojnurd
>KC106651_Iran__Bojnurd
>KC106652_Iran__Bojnurd
>KC138543_China
>KC138544_China
>KC138545_China
>KC138546_China
>KC211184_China__Shouguang_Shandong
>KC312655_China__AnHui_BengBu
>KC312656_China__AnHui_HeFei
>KC312657_China__HeBei_ShiJiazhuang
>KC312658_China__HeBei_TangShan
>KC312659_China__HuBei_WuHan

>KC312660_China__HuBei_WuHan
>KC312661_China__HeNan_ZhengZhou
>KC312662_China__JiangSu_NanJing
>KC312663_China__JiangSu_NanTong
>KC312664_China__ShanDong_liaoCheng
>KC312665_China__ShanDong_ShouGuang
>KC312666_China__Shandong_weifang
>KC312667_China
>KC312668_China__ShangHai
>KC312669_China__ShanXi_XiAn
>KC312670_China__ShanXi_XiAn
>KC312671_China__ZheJiang_HangZhou
>KC312672_China__ZheJiang_JiaXing
>KC312673_China__ZheJiang_NingBo
>KC428753_China__Kashi_Xinjiang
>KC677732_Japan
>KC702796_China__Changli_Heibei_province
>KC702797_China__Funing_Hebei_province
>KC702798_China__Songyuan_Jilin_province
>KC845301_Saudi_Arabia
>KC852147_China__Shandong_Shouguang
>KC852149_China__Shandong_Shouguang
>KC852150_China__Shandong_Shouguang
>KC852151_China__Shandong_Shouguang
>KC999844_China__Weifang_Shandong_Province
>KC999845_China__Weifang_Shandong_Province
>KC999846_China__Weifang_Shandong_Province
>KC999847_China__Weifang_Shandong_Province
>KC999848_China__Weifang_Shandong_Province
>KC999849_China__Weifang_Shandong_Province
>KC999850_China__Weifang_Shandong_Province
>KC999851_China__Shouguang
>KF225312_South_Korea
>KF229721_Oman__Sohar
>KF229722_Oman__Liwa
>KF229723_Oman__Barka
>KF229726_Oman__Barka
>KF435136_Saudi_Arabia
>KF435137_Saudi_Arabia
>KF444467_Saudi_Arabia
>KF477277_Venezuela
>KF533855_Costa_Rica
>KF533856_Costa_Rica

>KF533857_Costa_Rica
>KF561125_Saudi_Arabia
>KF612971_China
>KF906542_China
>KF990604_China__Guangdong
>KJ125410_China__Beijing
>KJ125411_China__Beijing
>KJ140787_China
>KJ140788_China
>KJ546418_China__Shandong_Shouguang
>NC_003803_SACMV
>NC_003828_Sardinia
>AF155806_SACMV
>AJ575560_SACMV_ZW
>KJ888031_SACMV_MG-MG529B2-11
>KJ887935_SACMV_MG-MG143A5-09
>KJ888044_SACMV_MG-MG571A1-11
>gi|685877403|gb|KJ887967.1| South African cassava mosaic virus isolate MG:MG250A1:10 segment A, complete sequence
>gi|685876722|gb|KJ887868.1| South African cassava mosaic virus isolate MG:MG50A1:06 segment A, complete sequence
>gi|685877946|gb|KJ888045.1| South African cassava mosaic virus isolate MG:MG573A1:11 segment A, complete sequence
>gi|685877932|gb|KJ888043.1| South African cassava mosaic virus isolate MG:MG570A3:11 segment A, complete sequence
>gi|685877835|gb|KJ888029.1| South African cassava mosaic virus isolate MG:MG521A1:11 segment A, complete sequence
>gi|685877828|gb|KJ888028.1| South African cassava mosaic virus isolate MG:MG516A1:11 segment A, complete sequence
>gi|685877758|gb|KJ888018.1| South African cassava mosaic virus isolate MG:MG471A1:11 segment A, complete sequence
>gi|685877710|gb|KJ888011.1| South African cassava mosaic virus isolate MG:MG411A1:11 segment A, complete sequence
>gi|685877703|gb|KJ888010.1| South African cassava mosaic virus isolate MG:MG411:06 segment A, complete sequence
>gi|685877626|gb|KJ887999.1| South African cassava mosaic virus isolate MG:MG369A2:11 segment A, complete sequence
>gi|685877605|gb|KJ887996.1| South African cassava mosaic virus isolate MG:MG347A1:11 segment A, complete sequence
>gi|685877226|gb|KJ887941.1| South African cassava mosaic virus isolate MG:MG175A3:09 segment A, complete sequence
>gi|685877144|gb|KJ887929.1| South African cassava mosaic virus isolate MG:MG137A3:09 segment A, complete sequence
>gi|685877055|gb|KJ887916.1| South African cassava mosaic virus isolate MG:MG122A2:09 segment A, complete sequence
>gi|685877048|gb|KJ887915.1| South African cassava mosaic virus isolate MG:MG120A3:09 segment

A, complete sequence
>gi|685876873|gb|KJ887890.1| South African cassava mosaic virus isolate MG:MG71A1:06 segment A, complete sequence
>gi|685876680|gb|KJ887862.1| South African cassava mosaic virus isolate MG:MG45A10:06 segment A, complete sequence
>gi|685877424|gb|KJ887970.1| South African cassava mosaic virus isolate MG:MG273A8:10 segment A, complete sequence
>gi|685877591|gb|KJ887994.1| South African cassava mosaic virus isolate MG:MG343A7:11 segment A, complete sequence
>gi|685877905|gb|KJ888039.1| South African cassava mosaic virus isolate MG:MG560A1:11 segment A, complete sequence
>gi|685877640|gb|KJ888001.1| South African cassava mosaic virus isolate MG:MG379A6:11 segment A, complete sequence
>gi|685876620|gb|KJ887853.1| South African cassava mosaic virus isolate MG:MG37A3:06 segment A, complete sequence
>gi|685877731|gb|KJ888014.1| South African cassava mosaic virus isolate MG:MG419B2:11 segment A, complete sequence
>gi|685877696|gb|KJ888009.1| South African cassava mosaic virus isolate MG:MG409A2:11 segment A, complete sequence
>gi|685877584|gb|KJ887993.1| South African cassava mosaic virus isolate MG:MG342B4:11 segment A, complete sequence
>gi|685877925|gb|KJ888042.1| South African cassava mosaic virus isolate MG:MG569A2:11 segment A, complete sequence
>gi|685877891|gb|KJ888037.1| South African cassava mosaic virus isolate MG:MG549A1:11 segment A, complete sequence
>gi|685877786|gb|KJ888022.1| South African cassava mosaic virus isolate MG:MG483A1:11 segment A, complete sequence
>gi|685877556|gb|KJ887989.1| South African cassava mosaic virus isolate MG:MG338A2:10 segment A, complete sequence
>gi|685877507|gb|KJ887982.1| South African cassava mosaic virus isolate MG:MG328A1:10 segment A, complete sequence
>gi|685877438|gb|KJ887972.1| South African cassava mosaic virus isolate MG:MG292:06 segment A, complete sequence
>gi|685877165|gb|KJ887932.1| South African cassava mosaic virus isolate MG:MG139A1:09 segment A, complete sequence
>gi|685877111|gb|KJ887924.1| South African cassava mosaic virus isolate MG:MG131A2:09 segment A, complete sequence
>gi|685877076|gb|KJ887919.1| South African cassava mosaic virus isolate MG:MG125A1:09 segment A, complete sequence
>gi|685876743|gb|KJ887871.1| South African cassava mosaic virus isolate MG:MG52C1:06 segment A, complete sequence
>gi|685876436|gb|KJ887826.1| South African cassava mosaic virus isolate MG:MG6A1:05 segment A, complete sequence
>gi|685877745|gb|KJ888016.1| South African cassava mosaic virus isolate MG:MG449A3:11 segment A, complete sequence
>gi|685877675|gb|KJ888006.1| South African cassava mosaic virus isolate MG:MG403A4:11 segment A, complete sequence
>gi|685877431|gb|KJ887971.1| South African cassava mosaic virus isolate MG:MG275A3:10 segment

A, complete sequence
>gi|685877062|gb|KJ887917.1| South African cassava mosaic virus isolate MG:MG123B3:09 segment A, complete sequence
>gi|685877884|gb|KJ888036.1| South African cassava mosaic virus isolate MG:MG545A3:11 segment A, complete sequence
>gi|685877863|gb|KJ888033.1| South African cassava mosaic virus isolate MG:MG533A1:11 segment A, complete sequence
>gi|685877821|gb|KJ888027.1| South African cassava mosaic virus isolate MG:MG512A2:11 segment A, complete sequence
>gi|685877814|gb|KJ888026.1| South African cassava mosaic virus isolate MG:MG503B1:11 segment A, complete sequence
>gi|685877563|gb|KJ887990.1| South African cassava mosaic virus isolate MG:MG339A1:10 segment A, complete sequence
>gi|685876396|gb|KJ887820.1| South African cassava mosaic virus isolate MG:MG1A3:06 segment A, complete sequence
>gi|685877654|gb|KJ888003.1| South African cassava mosaic virus isolate MG:MG400A4:11 segment A, complete sequence
>gi|685878008|gb|KJ888054.1| South African cassava mosaic virus isolate MG:MG594A1:11 segment A, complete sequence
>gi|685877807|gb|KJ888025.1| South African cassava mosaic virus isolate MG:MG492A4:11 segment A, complete sequence
>gi|685877772|gb|KJ888020.1| South African cassava mosaic virus isolate MG:MG474A6:11 segment A, complete sequence
>gi|685877647|gb|KJ888002.1| South African cassava mosaic virus isolate MG:MG398A1:11 segment A, complete sequence
>gi|685877549|gb|KJ887988.1| South African cassava mosaic virus isolate MG:MG337A1:10 segment A, complete sequence
>gi|685877542|gb|KJ887987.1| South African cassava mosaic virus isolate MG:MG336A5:10 segment A, complete sequence
>gi|685877528|gb|KJ887985.1| South African cassava mosaic virus isolate MG:MG334A3:10 segment A, complete sequence
>gi|685877233|gb|KJ887942.1| South African cassava mosaic virus isolate MG:MG176A3:09 segment A, complete sequence
>gi|685877192|gb|KJ887936.1| South African cassava mosaic virus isolate MG:MG146A1:09 segment A, complete sequence
>gi|685877118|gb|KJ887925.1| South African cassava mosaic virus isolate MG:MG133A8:09 segment A, complete sequence
>gi|685876887|gb|KJ887892.1| South African cassava mosaic virus isolate MG:MG73A1:06 segment A, complete sequence
>gi|685876729|gb|KJ887869.1| South African cassava mosaic virus isolate MG:MG51A1:06 segment A, complete sequence
>gi|685876660|gb|KJ887859.1| South African cassava mosaic virus isolate MG:MG44A1:06 segment A, complete sequence
>gi|685876443|gb|KJ887827.1| South African cassava mosaic virus isolate MG:MG7A33:05 segment A, complete sequence
>gi|685877125|gb|KJ887926.1| South African cassava mosaic virus isolate MG:MG134A2:09 segment A, complete sequence
>gi|685877717|gb|KJ888012.1| South African cassava mosaic virus isolate MG:MG414A1:11 segment

A, complete sequence
>gi|685877130|gb|KJ887927.1| South African cassava mosaic virus isolate MG:MG135A2:09 segment A, complete sequence
>gi|685878281|gb|KJ888094.1| South African cassava mosaic virus isolate MG:MG718A2:11 segment A, complete sequence
>gi|685878253|gb|KJ888090.1| South African cassava mosaic virus isolate MG:MG694A7:11 segment A, complete sequence
>gi|685878246|gb|KJ888089.1| South African cassava mosaic virus isolate MG:MG694A4:11 segment A, complete sequence
>gi|685877994|gb|KJ888052.1| South African cassava mosaic virus isolate MG:MG584A4:11 segment A, complete sequence
>gi|685877911|gb|KJ888040.1| South African cassava mosaic virus isolate MG:MG561A2:11 segment A, complete sequence
>gi|685877577|gb|KJ887992.1| South African cassava mosaic virus isolate MG:MG341A1:10 segment A, complete sequence
>gi|685877521|gb|KJ887984.1| South African cassava mosaic virus isolate MG:MG333A1:10 segment A, complete sequence
>gi|685876532|gb|KJ887840.1| South African cassava mosaic virus isolate MG:MG20A1:06 segment A, complete sequence
>gi|685877800|gb|KJ888024.1| South African cassava mosaic virus isolate MG:MG491A8:11 segment A, complete sequence
>gi|685877793|gb|KJ888023.1| South African cassava mosaic virus isolate MG:MG490A3:11 segment A, complete sequence
>gi|685877779|gb|KJ888021.1| South African cassava mosaic virus isolate MG:MG475A3:11 segment A, complete sequence
>gi|685877765|gb|KJ888019.1| South African cassava mosaic virus isolate MG:MG473A2:11 segment A, complete sequence
>gi|685877751|gb|KJ888017.1| South African cassava mosaic virus isolate MG:MG467A1:11 segment A, complete sequence
>gi|685877738|gb|KJ888015.1| South African cassava mosaic virus isolate MG:MG420B3:11 segment A, complete sequence
>gi|685877417|gb|KJ887969.1| South African cassava mosaic virus isolate MG:MG262A2:10 segment A, complete sequence
>gi|685877158|gb|KJ887931.1| South African cassava mosaic virus isolate MG:MG138B1:09 segment A, complete sequence
>gi|685877090|gb|KJ887921.1| South African cassava mosaic virus isolate MG:MG127A1:09 segment A, complete sequence
>gi|685876606|gb|KJ887851.1| South African cassava mosaic virus isolate MG:MG32B1:06 segment A, complete sequence
>gi|685877069|gb|KJ887918.1| South African cassava mosaic virus isolate MG:MG124A3:09 segment A, complete sequence
>gi|685878302|gb|KJ888097.1| South African cassava mosaic virus isolate MG:MG730A4:11 segment A, complete sequence
>gi|685878288|gb|KJ888095.1| South African cassava mosaic virus isolate MG:MG729A3:11 segment A, complete sequence
>gi|685878066|gb|KJ888063.1| South African cassava mosaic virus isolate MG:MG624A4:11 segment A, complete sequence
>gi|685877535|gb|KJ887986.1| South African cassava mosaic virus isolate MG:MG335A6:10 segment

A, complete sequence
>gi|685877362|gb|KJ887961.1| South African cassava mosaic virus isolate MG:MG238A1:10 segment A, complete sequence
>gi|685877301|gb|KJ887952.1| South African cassava mosaic virus isolate MG:MG209A2:09 segment A, complete sequence
>gi|685877178|gb|KJ887934.1| South African cassava mosaic virus isolate MG:MG142A3:09 segment A, complete sequence
>X76319_Israel
>Z83256_EACMV_Tanzania
