# Supplementary material for: Vector-Enabled Metagenomic (VEM) Surveys Using Whiteflies (Aleyrodidae) Reveal Novel Begomovirus Species in the New and Old Worlds
Source: Viruses. 2015 Oct 26;7(10):5553–70. doi: 10.3390/v7102895 (PMC4632403; doi:10.3390/v7102895)
Supplement: Supplementary File 1 [file viruses-07-02895-s001.zip › SupplementalTable_S1.pdf]

Supplemental Table S1. Genomic features of begomoviruses detected in whiteflies.

Genome[a]	Genome Size	CP motif[b]	DNA-B MP motif[c]	CR (length/ % identity)[d]
VEM Squash leaf curl virus DNA-A [California squash 2012 Contig 2]	2638	SWRLMAGT		228/95%
VEM Squash leaf curl virus DNA-B [California squash 2012 Contig 1]	2641		RCNIDLHY	
VEM Melon chlorotic leaf curl virus DNA-A [Guatemala squash 2012 Contig 6]	2662	PWRLLAGT		198/93%
VEM Melon chlorotic leaf curl virus DNA-B [Guatemala squash 2012 Contig 13]	2630		RCNIDLHY	
VEM Tomato severe leaf curl virus DNA-A [Guatemala squash 2012 Contig 17]	2593	PWRLMAGT		
VEM Sida golden mosaic Honduras virus 1a DNA-A [Guatemala squash 2012]	2608	PWRAMPGI		160/87%
VEM Sida golden mosaic Honduras virus 1b DNA-A [Guatemala squash 2012]	2608	PWRAMPGT		
VEM Sida golden mosaic Honduras virus 1 DNA-B [Guatemala squash 2012]	2557		RCNIDLHY	
VEM begomovirus 1a DNA-A [Guatemala squash 2012]	2610	PWRLVETL		
VEM begomovirus 1b DNA-A [Guatemala squash 2012]	2610	PWRLVETL		
VEM begomovirus 1c DNA-A [Guatemala squash 2012]	2609	PWRLVETL		
VEM begomovirus 2 DNA-A [Guatemala squash 2012]	2590	PWRPMAGT		
VEM begomovirus 3a DNA-A [Guatemala squash 2012]	2629	PWRTMAGT		223/99%
VEM begomovirus 3b DNA-A [Guatemala squash 2012]	2628	PWRTMAGT		
VEM begomovirus 3a DNA-B [Guatemala squash 2012]	2580		RCNIDLHY	
VEM begomovirus 3b DNA-B [Guatemala squash 2012]	2580		RCNIDLHY	
VEM begomovirus 4 DNA-A [Guatemala squash 2012]	2608	SWRLMAGT		
VEM Tomato severe leaf curl virus DNA-A [Guatemala tomato 2012 Contig 1]	2593	PWRLMAGT		
VEM Tomato mosaic Havana virus DNA-A [Guatemala tomato 2012 Contig 18]	2619	PWRTMAVT		184/96%
VEM Tomato mosaic Havana virus DNA-B [Guatemala tomato 2012 Contig 12]	2606		RCNIDLHY	
VEM Squash leaf curl virus DNA-A [Israel squash 2011]	2637	PWRLMAGT		228/95%
VEM Squash leaf curl virus DNA-B [Israel squash 2011 Contig 1]	2608		RCNIDLHY	
VEM Cotton leaf curl Gezira virus DNA-A [Israel squash 2011]	2758			
VEM Sweet potato leaf curl virus [Puerto Rico tomato 2010]	2804			
VEM begomovirus 5a DNA-A [Puerto Rico tomato 2010]	2606	SWRSMAGT		150/98%
VEM begomovirus 5b DNA-A [Puerto Rico tomato 2010]	2606	SWRSMAGT		
VEM begomovirus 5c DNA-A [Puerto Rico tomato 2010]	2607	SWRSLAGT		
VEM begomovirus 5d DNA-A [Puerto Rico tomato 2010]	2607	SWRSLAGT		
VEM begomovirus 5e DNA-A [Puerto Rico tomato 2010]	2606	SWRSMAGT		
VEM begomovirus 5 DNA-B [Puerto Rico tomato 2010]	2586		RCNIDLHY	
VEM begomovirus 6b DNA-B [Puerto Rico tomato 2010]	2574		RCNIDLHY	
VEM begomovirus 6 DNA-A [Puerto Rico pumpkin 2010]	2638	PWRSMAGP		162/88%
VEM begomovirus 6a DNA-B [Puerto Rico pumpkin 2010]	2572		RCNIDLHY	

Genome[a]	Genome Size	CP motif[b]	DNA-B MP motif[c]	CR (length/ % identity)[d]
VEM Sweet potato leaf curl virus [Puerto Rico pumpkin 2010]	2788			
VEM Macroptilium mosaic Puerto Rico virus DNA-A [Puerto Rico pumpkin 2010 Contig 5]	2616	PWRSSAGT		160/94%
VEM Macroptilium mosaic Puerto Rico virus DNA-B [Puerto Rico pumpkin 2010 Contig 1]	2570		RCNIDLHY	
VEM begomovirus 5f DNA-A [Puerto Rico eggplant 2010]	2607	SWRSLAGT		
VEM Sweet potato leaf curl virus [Puerto Rico eggplant 2010]	2823			
VEM Sweet potato leaf curl virus [Spain bean 2011]	2829			
VEM Sweet potato leaf curl virus [Spain squash 2011]	2765			
VEM begomovirus 7a [Spain tomato 2011]	2769			
VEM begomovirus 7b [Spain tomato 2011]	2767			
VEM begomovirus 7c [Spain tomato 2011]	2767			
VEM begomovirus 7d [Spain tomato 2011]	2767			
VEM begomovirus 7e [Spain tomato 2011]	2767			
VEM begomovirus 7f [Spain tomato 2011]	2780			
VEM begomovirus 7g [Spain tomato 2011]	2780			
VEM begomovirus 7h [Spain weed 2011]	2769			
VEM begomovirus 7i [Spain weed 2011]	2769			
VEM begomovirus 7j [Spain weed 2011]	2769			
VEM begomovirus 7k [Spain weed 2011]	2767			
VEM Tomato yellow leaf curl virus-[Almeria] 1 [Spain weed 2011 Contig 35]	2781			
VEM Tomato yellow leaf curl virus-[Almeria] 1a [Spain weed 2011 Contig 1]	2781			

[a]Novel genomes sharing <91% identity with known begomovirus genomes were named 'VEM begomoviruses'. Genomes that were not PCR-verified include the word 'Contig' within brackets.
[b]Conserved motif found within the N-terminus of capsid proteins (CP) encoded by New World bipartite begomoviruses.
[c]Conserved motif found within the majority of movement proteins (MP) encoded by New World bipartite begomoviruses.
[d]Length and percent identity between the common region (CR) sequences of putative cognate DNA-A and DNA-B components of bipartite begomoviruses.
